# Correction: FoxO3a Modulates Hypoxia Stress Induced Oxidative Stress and Apoptosis in Cardiac Microvascular Endothelial Cells

**DOI:** 10.1371/annotation/1c419628-f1b5-45de-9f8a-43f834309ebb

**Published:** 2013-12-16

**Authors:** Shenwei Zhang, Yilin Zhao, Ming Xu, Li Yu, Yujie Zhao, Jianghong Chen, Yiqiang Yuan, Qiangsun Zheng, Xiaolin Niu

Yiqiang Yuan and Qiangsun Zheng, the seventh and eighth authors, respectively, should be indicated as being Corresponding Authors for the article. Both of the authors share the same email address as the Corresponding Author already indicated. In addition, the affiliation for the fifth author is incorrect. Yujie Zhao is not affiliated with institution number 1, but with institution number 2, Department of Cardiology, Tangdu Hospital, the Fourth Military Medical University, Xi’an.

